# Postural Orthostatic Tachycardia Syndrome Presenting With Recurrent Syncope After Cervical Spinal Cord Injury

**DOI:** 10.1002/ccr3.72181

**Published:** 2026-02-28

**Authors:** Yonghong Liu, Boyan Fang, Qiaoxia Zhen

**Affiliations:** ^1^ Department of Neurology Beijing Rehabilitation Hospital Affiliated to Capital Medical University Beijing China

**Keywords:** postural orthostatic tachycardia syndrome, postural syncope, rehabilitation, spinal cord injury

## Abstract

Syncope is a manifestation of autonomic dysfunction after high spinal cord injury. However, it is rarely reported as a feature of postural orthostatic tachycardia syndrome (POTS) after spinal cord injury. This case report describes a male in his 50s suffering from C2 spinal cord injury who developed recurrent postural syncope post‐injury. These events were characterized by orthostatic tachycardia upon standing and could even be induced by seated head‐tilt maneuvers, fulfilling the diagnostic criteria for POTS. These patients have substantial risks of fall‐related morbidity. Heart rate variability and sympathetic skin response assessments help elucidate the underlying autonomic pathophysiological mechanisms. Syncope may be the predominant symptom during transfers, standing, or seated head‐tilt positioning. Notably, documenting heart rate fluctuations during transient syncopal events is challenging. Infections constitute established triggers for syncope. Comprehensive management strategies may achieve complete resolution of syncopal episodes.

## Key Clinical Message

Postural orthostatic tachycardia syndrome can occur after high cervical spinal cord injury, even in patients with an American Spinal Injury Association Impairment Scale grade of D. Recurrent syncope during rehabilitation may be the predominant manifestation, and both heart rate variability and sympathetic skin response may facilitate and support identification of autonomic dysfunction. Comprehensive rehabilitation management demonstrates favorable therapeutic outcomes.

## 1 Background

High spinal cord injury (HSCI) (above T6) disrupts autonomic regulation of the cardiovascular system. Sympathetic pathways above T6 traverse the spinal cord, whereas parasympathetic fibers course within the vagus nerve. Consequently, HSCI acutely impairs sympathetic regulation. Syncope is one of the manifestations of autonomic dysfunction following HSCI [1, 2, 3], with orthostatic hypotension (OH) [2], and vasovagal syncope (VVS) [4] representing common etiologies attributed to sympathetic hypoactivity and unopposed parasympathetic signaling.

Only a few case reports describe postspinal cord injury (SCI) postural orthostatic tachycardia syndrome (POTS) presenting with syncope [5]. The pathophysiology remains incompletely elucidated. According to current evidence, POTS is a subtype of a cardiovascular autonomic dysregulation, characterized by three distinct mechanisms: hypovolemic, hyperadrenergic and neuropathic subtypes [6]. Proposed mechanisms include disorders of autonomic cardiovascular regulation, involving both abnormally increased sympathetic activity and peripheral sympathetic denervation leading to central hypovolemia. According to this hypothesis, inadequate sinus tachycardia can be explained by increased central sympathetic outflow, whereas heart rate (HR) elevation due to venous pooling and reduced preload leading would be explained by peripheral sympathetic denervation. Both pathomechanisms acting together result in characteristic postural tachycardia [7].

Notably, OH remains relatively common but occurs in only 11.6% of cervical spine surgery patients [2]. In contrast, syncope directly caused by POTS is exceptionally rare, particularly in patients with American Spinal Injury Association Impairment Scale grade of D(AIS‐D). Among 150 SCI patients treated at our neurorehabilitation center, only this case exhibited POTS‐related syncope, confirming its low incidence. Therefore, substantial fall‐related morbidity risks necessitate heightened clinical vigilance.

Compared with previously reported cases of POTS secondary to SCI [5], the present case involved a patient with C2 AIS‐D injury, who had more preserved motor and sensory functions. Notably, this is the first report to systematically incorporate HRV and SSR assessments, and explicitly identify infection as a precipitating factor. This study thus fills the gap in clinical data regarding POTS complicated with syncope in patients with AIS‐D SCI.

## 2 Case Presentation

**FIGURE 1:**
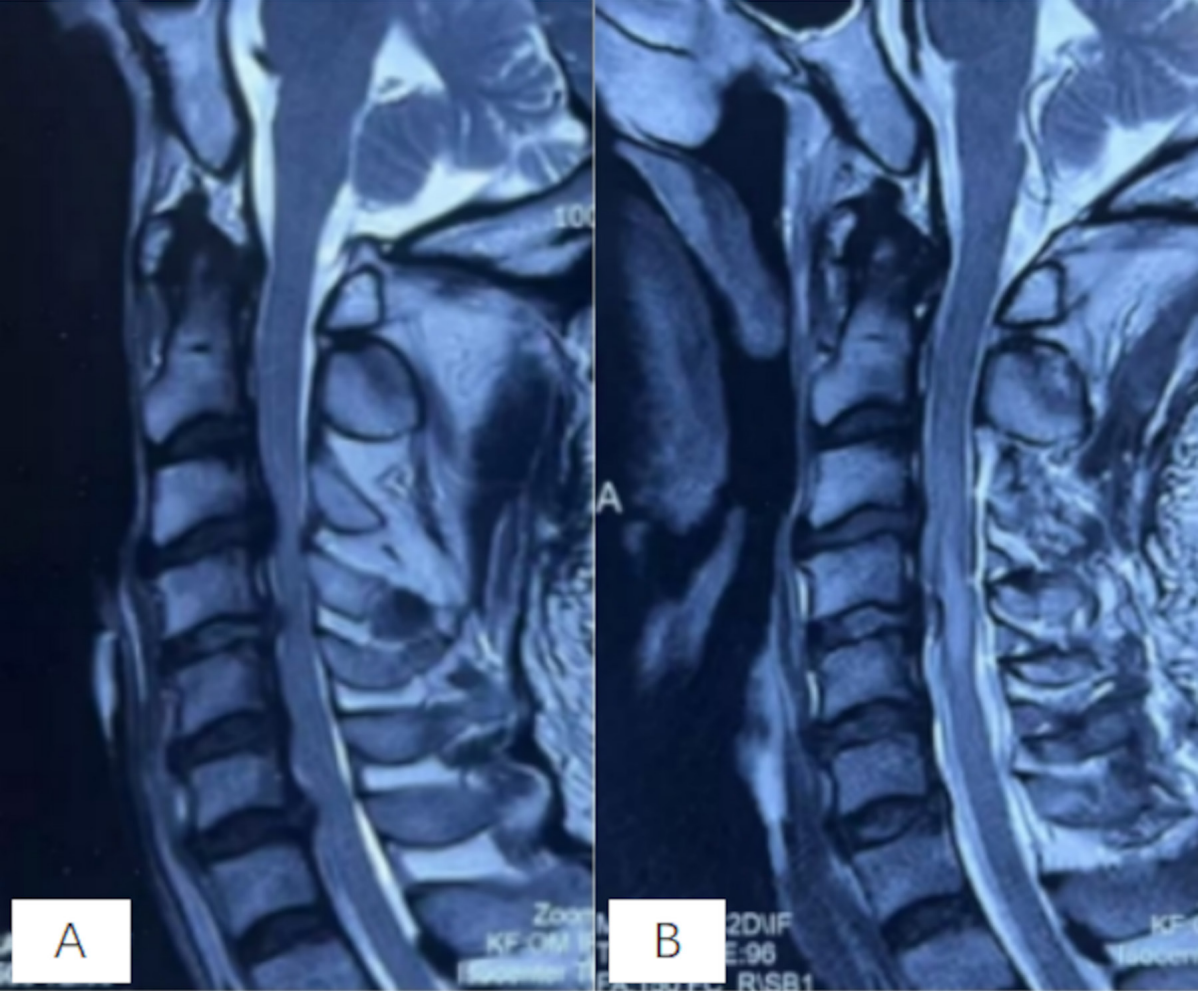
Radiographic evaluation of the cervical spinal cord. (A) Preoperative sagittal T2‐weighted MRI showing spinal canal stenosis at C3–C6 with cord compression. (B) Postoperative sagittal T2‐weighted MRI following posterior cervical decompression, showing successful expansion of the spinal canal and relief of the previous cord compression.

A male in his 50s presented with a history of cervical disc degeneration and mild protrusion; notably, he had remained asymptomatic with no prior limb numbness, muscle atrophy, weakness, or dizziness. On the day of injury, after maintaining a seated, flexed neck posture for over an hour while using a smartphone, the patient experienced neck stiffness and applied a percussive massage device to his neck. This was immediately followed by mild posterior cervical pain. During transit to the hospital, he developed progressive tetraparesis. Within 2 h, he presented with complete paralysis of the right limbs and only minimal voluntary movement on the left, accompanied by sensory loss in all four extremities. Urgent magnetic resonance imaging (MRI) revealed ossification of the posterior longitudinal ligament, spinal stenosis, and acute SCI (Figure 1A). Posterior cervical decompression (C3–C6) was performed on post‐injury day 2 (Figure 1B).

He was transferred to our rehabilitation unit 17 days after injury. At admission, antibiotic therapy was initiated for pulmonary and urinary tract infections (UTI). Duloxetine was prescribed to manage post‐traumatic anxiety. Notably, the patient had a history of hypertension requiring chronic antihypertensive medication; however, following the SCI, his blood pressure (BP) trended towards the normal range, and the medication was discontinued. No prior cardiac history was documented. His examination findings were motor and sensory levels bilaterally at C2; neurological level of injury at C2; Key muscle group strength was grade 4 on the left and ranged from 0 to 3 on the right; American Spinal Injury Association Impairment Scale (AIS)‐D. The initial Spinal Cord Injury Independence Measure (SCIM) score was 10.

## 3 Investigations

Pulmonary infection resolved after antibiotic treatment. Twenty‐four‐hour ambulatory blood pressure (BP) monitoring showed a mean of 154/90 mmHg, which prompted resumption of antihypertensive therapy. A structured rehabilitation protocol, encompassing physical rehabilitation and activities of daily living training aimed at improving endurance, respiratory function, and extremity muscle strength, was initiated on hospital Day 2. This program also incorporated acupuncture and interferential current therapy as adjuvant modalities. Motor function showed marked improvement thereafter: by 4 weeks post‐injury, he was able to stand with minimal unilateral assistance and walk wearing a cervical collar, without episodes of syncope.

At 10 weeks post‐injury, the patient experienced syncope lasting approximately 10 s during a wheelchair‐to‐bed transfer. Actually, prior to this episode, there were two shorter instances of suspected fainting lasting approximately 2–3 s; however, both the patient and the caregiver were uncertain if these were true syncopal episodes. The recovery was spontaneous and accompanied by facial flushing and diaphoresis. Episodes recurred frequently (1–5/day), occasionally with mild right limb tremors, severely disrupting rehabilitation. Most syncope occurred during transfers; briefer episodes were triggered by seated forward head tilt. Orthostatic BP and heart rate (HR) measurements (Table 1) revealed that syncopal episodes were consistently associated with marked HR elevations rather than BP fluctuations. For instance, during a syncope‐associated transfer, orthostatic testing demonstrated an HR increase of 31 bpm in the absence of significant BP changes. Although a single instance of significant BP drop (31/12 mmHg) with a 20 bpm HR elevation was recorded during a standing test, this occurred during post‐syncope recovery and was notably asymptomatic. Across multiple longitudinal measurements, this was the sole instance of significant BP reduction recorded.

**TABLE 1:** Orthostatic BP/HR measurements.

Posture	BP (mmHg)	HR (bpm)	Clinical status
Wheelchair sitting	137/91	93	Asymptomatic
During wheelchair‐stand‐bed transfer	136/89	124	5‐s syncope
Supine	149/94	92	Asymptomatic
Standing (1 min)	118/82	112	Asymptomatic
Seated	142/93	94	Asymptomatic

Abbreviations: BP, blood pressure; bpm, beats per minute; HR, heart rate.

**FIGURE 2:**
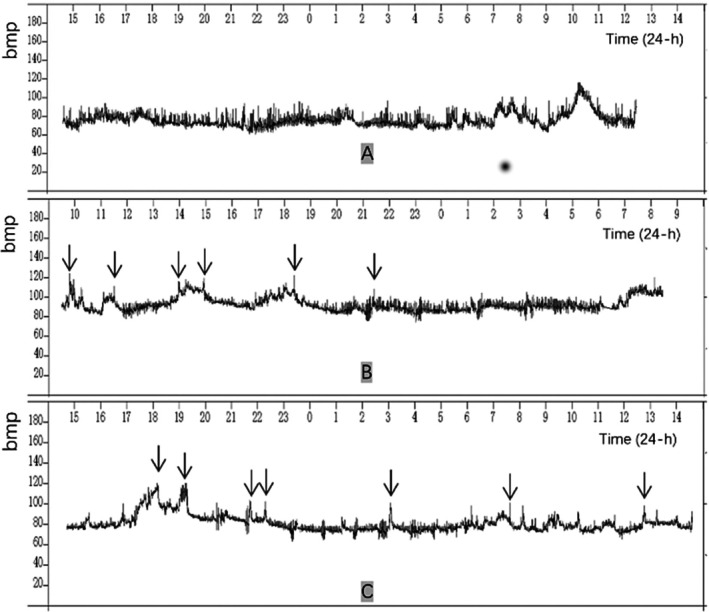
Serial 24‐h Holter monitoring of HR fluctuations. (A) Admission: Mean HR 75 bpm (range: 60–114 bpm) and SDNN 75 ms. (B) Symptomatic phase (post‐syncope onset): Mean HR increased to 92 bpm (range: 73–121 bpm) with a decreased SDNN of 48 ms; arrows indicate daytime tachycardic episodes. No syncope occurred during this specific recording window. (C) Posttreatment (15 weeks post‐injury): Mean HR 79 bpm (range: 62–118 bpm) and SDNN 72 ms. Despite persistent tachycardic episodes (arrows), syncopal frequency was reduced to approximately once per week, and no syncope occurred during this recording. bpm, beats per minute; HR, heart rate; SDNN, standard deviation of normal‐to‐normal RR intervals.

Following the onset of syncope, 24‐h Holter monitoring revealed significantly reduced heart rate variability (HRV), with a standard deviation of normal‐to‐normal RR intervals (SDNN) of 48 ms (reference range: 141 ± 39 ms). Abrupt HR increases to 121 bpm during positional changes occurred without symptoms. The evolution of these parameters across the patient's clinical course—from admission through the symptomatic peak to posttreatment recovery—is detailed in Figure 2.

**FIGURE 3:**
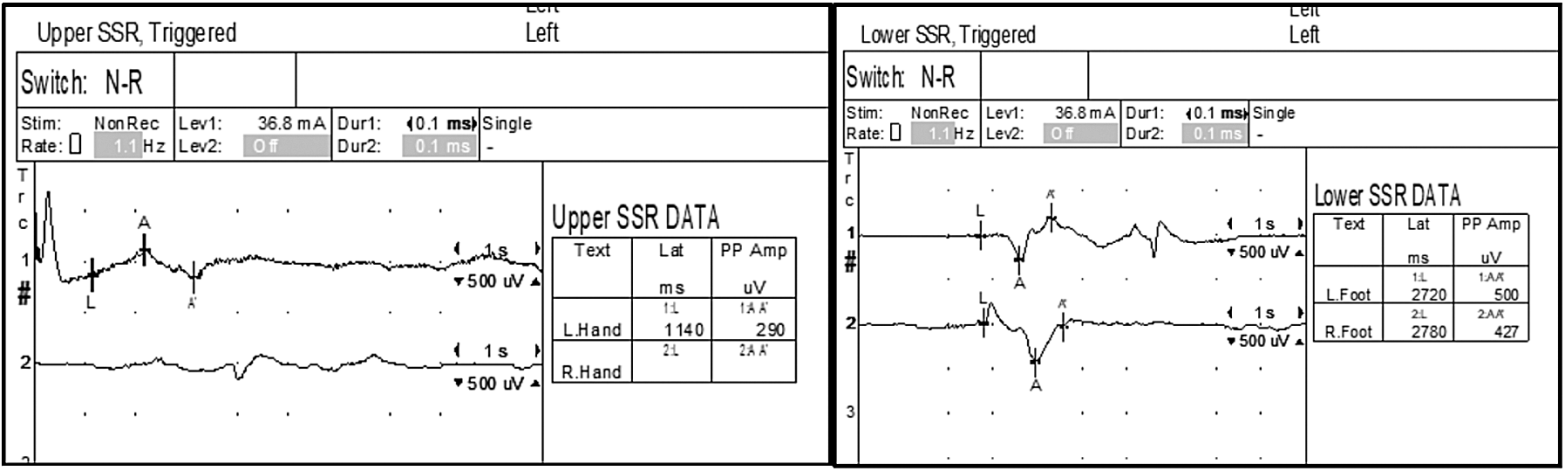
SSR Results (A) Upper limbs (B) Lower limbs.

Sympathetic Skin Response (SSR) demonstrated mildly abnormal sympathetic function in the left upper limb (reduced amplitude), lack of reproducible response in the right upper limb, and normal responses in both lower limbs (Figure 3).

Follow‐up cervical MRI showed no SCI progression. Vascular ultrasounds of the head and neck, magnetic resonance angiography, echocardiography, electroencephalography, complete blood count, renal/liver function, electrolytes, BNP, cardiac enzymes, thyroid function, and supine renin/aldosterone—all were normal. Recurrent syncope occurred exclusively during positional changes, without associated tonic–clonic movements, headache, chest pain, palpitations, urinary or bowel retention, or emotional triggers. Carotid sinus massage and Valsalva maneuver provocation tests were negative.

Clinically, syncope frequently occurred almost immediately (within 15 s) during wheelchair‐to‐bed transfers or seated head‐tilting maneuvers. Synchronized HR and BP monitoring during these episodes consistently revealed a rapid HR increase from 80–95 bpm to 110–125 bpm (HR rise ≥ 30 bpm), which typically lasted 3–10 s before returning to baseline. No concurrent BP drop was recorded during these syncopal events. Furthermore, the patient frequently exhibited asymptomatic orthostatic tachycardia (HR rise ≥ 25 bpm) during routine transfers, while resting HR remained consistently < 100 bpm. A formal head up tilt table test was not performed. However, the diagnosis was substantiated through repeated bedside orthostatic testing that captured the diagnostic criteria during symptomatic positional transitions. Clinical hypovolemia was excluded based on the patient's adequate fluid intake and normal urine output. Additionally, medication‐induced tachycardia was ruled out, as the patient was only taking probiotics, mosapride, and digestive enzymes, with no history of using sympathomimetics, vasodilators, or other HR‐modifying agents.

Comprehensive evaluation confirmed that the patient met the current diagnostic criteria for POTS: (1) an HR increment of > 30 bpm within 5 min of standing or positional change; (2) HR reaching ≥ 120 bpm during orthostatic stress; (3) persistent orthostatic symptoms; and (4) the absence of other overt causes of tachycardia [8]. While some criteria suggest excluding heart rate increases that occur within the first 60 s of standing to account for initial physiological compensation [9], the presence of frank syncope in this patient suggests a profound failure of autonomic stabilization rather than a normal compensatory response.

## 4 Differential Diagnosis

Differential diagnoses included OH, VVS, inappropriate sinus tachycardia (IST), psychogenic pseudosyncope (PPS), epilepsy, acute cerebrovascular disease, and primary cardiac disease. Although BP dropped during positional changes, syncope was not due to OH. This is because episodes also occurred during head‐tilt positioning, which is pathophysiologically incompatible with OH. VVS was excluded because of the absence of bradycardia or hypotension during sustained upright posture. Critically, the patient had no history of cardiac disease, echocardiography revealed no structural abnormalities, and the resting HR consistently stayed below 100 bpm, ruling out cardiogenic IST or primary cardiac disease. Normal brain MRI and electroencephalography results did not support acute cerebrovascular disease or epilepsy. Regarding PPS, although the patient's scores on the Hamilton Anxiety Rating Scale [10] and Hamilton Depression Rating Scale‐17 [11] suggested mild anxiety and possible depression, several clinical features argued against this diagnosis. PPS typically involves prolonged episodes (lasting minutes to over half an hour) with stable heart rate and blood pressure, and is often triggered by emotional distress or witnessing medical procedures. In this case, syncope was strictly orthostatic. Furthermore, the patient remained syncope‐free during the early admission period when emotional fluctuations were most intense, and syncopal events only emerged later as physical mobility improved and mood stabilized, further differentiating it from PPS.

## 5 Treatment and Outcomes

**FIGURE 4:**
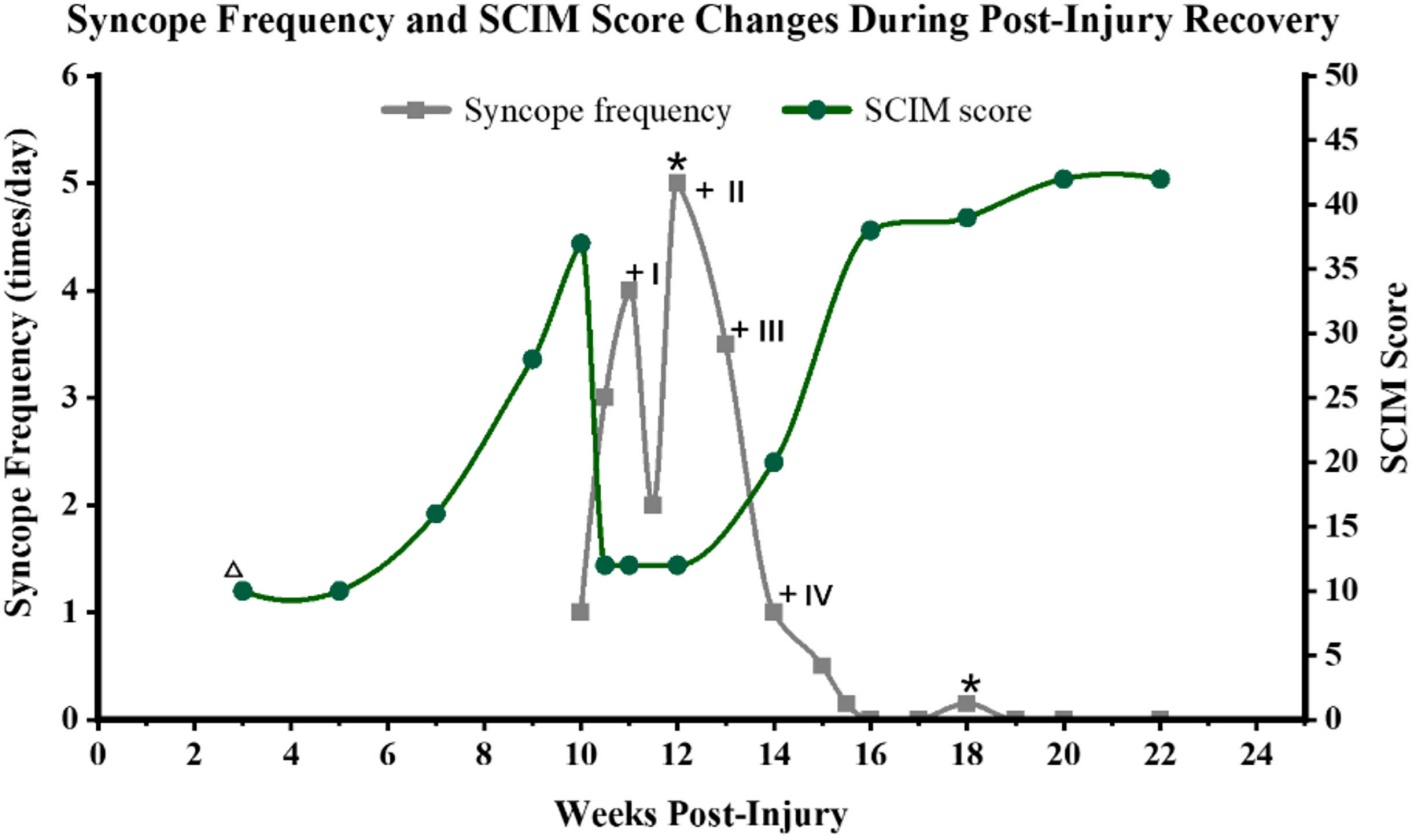
Timeline of syncope frequency, interventions, and functional recovery. The chart illustrates the changes in syncope frequency and SCIM scores throughout the recovery period. △: Structured rehabilitation. *, Urinary tract infection events; + I, Initiation of external counterpulsation therapy and strictly graded postural transitions; + II, Anti‐infection therapy, application of an abdominal binder and compression stockings; + III, Introduction of Metoprolol; + IV, Implementation of pre‐transfer lateral head movements; SCIM, Spinal Cord Injury Independence Measure.

Upon admission, the patient followed a structured rehabilitation protocol. Before the onset of syncope, his motor function had improved significantly, enabling him to walk slowly for approximately 20 m with crutches (SCIM score: 37). After syncopal episodes emerged, management was intensified (Figure 4). In addition to the ongoing rehabilitation, thrice‐weekly external counterpulsation therapy and a strictly graded postural transition protocol were implemented. This transition protocol consisted of: (a) Stepwise bed elevation (30° → 60° → 90° over 5 min), (b) Sustained sitting at bedside (minimum 3 min), and (c) Wheelchair transfer preparation (minimum 2 min). Total transition duration ≥ 10 min. Following these measures, syncope frequency decreased to 1–3 episodes daily.

At Week 12, a UTI exacerbation with fever increased syncope frequency to 5 episodes daily. Management included aggressive infection control, combined with the application of an abdominal binder and compression stockings during transfers and continued counterpulsation; syncope frequency subsequently declined to 3–4 episodes daily.

During sitting and standing transfers, it was observed that the patient frequently experienced rapid heart rate increases lasting 3–10 s, with the heart rate typically surging from 80–90 bpm to 100–125 bpm. A cardiology consultation was subsequently requested, following which Metoprolol (12.5 mg twice daily) was initiated. Metoprolol was considered a rational choice as the patient's clinical presentation was dominated by sympathetic hyperactivity and marked tachycardia with relatively stable blood pressure. Midodrine was not used due to the absence of hypotension or dizziness, and fludrocortisone was avoided as there was no evidence of hypovolemia. Since the patient showed significant improvement and stabilized heart rate fluctuations after Metoprolol, alternative agents such as ivabradine were not pursued. Following this, syncope decreased to 1–2 episodes daily.

Around Week 14, the patient noted that pre‐transfer lateral head movements for several minutes further reduced symptoms. Syncope frequency subsequently declined to once weekly by Weeks 15–16, with complete resolution between Weeks 16 and 18. During this period, the HR increment during supine‐to‐standing transfers stabilized at 10–20 bpm. Follow‐up HRV analysis demonstrated that the SDNN had increased to 72 ms. Although a second UTI at Week 18 triggered two brief episodes, aggressive anti‐infective therapy prevented further recurrence.

## 6 Follow‐Up

At over 5 months post‐injury during ongoing rehabilitation, the patient could walk independently for about 200 m and had partial bladder control, voiding approximately 200 mL when bladder volume exceeded 300 mL. The SCIM score reached 42 at 22 weeks post‐injury.

## 7 Discussion

POTS may develop due to autonomic dysfunction. It is characterized by an exaggerated increase in HR upon postural changes, defined as a HR rise of ≥ 30 bpm or an absolute rate of ≥ 120 bpm, without OH (systolic BP drop < 20 mmHg) [12]. HSCI can induce autonomic dysfunction, in which syncope represents a clinical manifestation. Syncope typically occurs during standing or head‐up tilt positioning [13]. Post‐SCI POTS orthostatic symptoms may include chest discomfort, palpitations, lightheadedness, visual disturbances, headache, dyspnea, fatigue, tremor, nausea, vomiting, or syncope, without hypotension or alternative tachycardic etiologies [5]. This patient demonstrated POTS with syncope as the predominant manifestation during transfers/standing or seated head tilting. Syncopal events were sometimes accompanied by mild limb tremors, fatigue, and appetite suppression, but without dizziness, headache, palpitations, or dyspnea. Although asymptomatic tachycardia occurred during other positional changes, this variable symptomatology complicates clinical recognition. Head‐up tilt testing contributed to diagnostic confirmation [12].

The pathophysiology in this case supports a hypothesis of neuropathic POTS following HSCI. This is suggested by: (1) abnormal SSR and reduced HR variability (SDNN < 70 milliseconds) indicating sympathetic dysregulation [14], (2) absence of anemia/hypovolemia, (3) normal supine renin/aldosterone levels. However, it must be emphasized that in a single case, the linkage between reduced HRV/SSR abnormalities and neuropathic POTS remains hypothesis‐supporting rather than definitive, as these parameters can be significantly influenced by factors such as pain, anxiety, medications, and physical deconditioning.

To achieve a more definitive phenotyping, further specialized testing would be warranted. For instance, Quantitative Sudomotor Axon Reflex Testing or a Thermoregulatory Sweat Test could explicitly clarify the presence of postganglionic sympathetic cholinergic fiber dysfunction. Furthermore, while our patient lacked clinical indicators of hypovolemia (e.g., anemia or insufficient intake), a 24‐h urine sodium concentration is recommended for a gold standard assessment of volume status. To exclude a hyperadrenergic phenotype, measurement of upright plasma norepinephrine levels would be necessary. As emphasized in clinical guidelines, POTS phenotypes are not distinguishable based on symptoms alone; if precise phenotyping is sought, comprehensive physiological testing is essential [6].

POTS is a heterogeneous and multifactorial disorder involving both peripheral and central autonomic pathways. In neuropathic POTS, peripheral autonomic injury—primarily small‐fiber neuropathy—is a core mechanism. This peripheral denervation leads to impaired vasoconstriction in the lower extremities, resulting in excessive venous pooling during orthostatic stress, which “passively” triggers compensatory tachycardia. Conversely, central autonomic dysfunction following HSCI may stem from abnormalities in regulatory regions such as the hypothalamus or brainstem, or hyperresponsiveness of the adrenal medulla. The loss of descending inhibitory control leads to “active” sympathetic overexcitation [6, 11, 15].

To further contextualize our findings, we compared this case with a similar report by Yadav et al. (Table 2) [5]. While both cases involved spinal cord injury and demonstrated significant orthostatic tachycardia, the clinical presentations differed. In the case by Yadav et al. (T7, AIS‐B), symptoms such as fatigue and presyncope were elicited upon reaching a 60‐degree head tilt without OH. In contrast, our patient (C2, AIS‐D) experienced complete syncope triggered during active wheelchair‐to‐bed transfers, standing, or seated forward head tilting. Notably, our case highlights systemic infection as a critical provocative factor for autonomic instability. While both management strategies utilized Metoprolol, our protocol integrated additional interventions.

**TABLE 2:** Clinical comparison between the our case and Yadav et al. [5].

Feature	Yadav et al. (*Cureus*, 2023)	Our patient
NLI (AIS grading)	T7 (AIS‐B)	C2 (AIS‐D)
Etiology	Trauma (fall from height)	Degeneration + Minor trauma
POTS symptoms	Fatigue, dizziness, headache, palpitations, and presyncope; triggered after achieving 60° head tilt on a tilt table	Complete syncope, fatigue, facial flushing, and limb tremors; triggered during wheelchair‐to‐bed transfers, standing or seated forward head tilt
HR increase	35–40 bpm; No OH	10–40 bpm; Occasional asymptomatic OH
Triggering factors	Not mentioned	Systemic infection (UTI)
Diagnosis	Tilt‐table test (60°)	Bedside orthostatic testing, HRV, and SSR
Treatment	Metoprolol; 3 L water; Abdominal binder/stockings	Metoprolol; 2 L water; Abdominal binder/stockings
Specific interventions	Structured rehabilitation protocol	Structured rehabilitation protocol, external counterpulsation, graded transitions, lateral head movements
Outcome	Symptoms resolved in 5 days; Tachycardia settled within 1 week; FIM 48 → 82	Syncope resolved by Week 18 post‐injury; SCIM 10 → 42

Abbreviations: AIS, American Spinal Injury Association Impairment Scale; C, cervical; FIM, functional independence measure; HR, heart rate; HRV, heart rate variability; L, Liter; NLI, neurological level of injury; OH, orthostatic hypotension; POTS, postural orthostatic tachycardia syndrome; SCIM, Spinal Cord Injury Independence Measure; SSR, sympathetic skin response; T, thoracic; UTI, urinary tract infection.

In this case, UTIs consistently triggered syncopal episodes; symptoms resolved after infection control but recurred during subsequent exacerbations, highlighting infections as significant exacerbators of autonomic instability [10]. Cytokine release or systemic inflammation triggered by infection may play a critical role. Inflammatory mediators may directly affect autonomic ganglia and neurons, induce autoimmune responses against the autonomic nervous system (e.g., the production of anti‐G‐protein coupled receptor antibodies), or impair brainstem cardiovascular regulatory centers and norepinephrine transporter function. These mechanisms collectively disrupt heart rate and blood pressure regulation, precipitating the syncopal episodes observed in our patient during UTIs exacerbations [16].

Therapeutic interventions for post‐SCI POTS remain limited in the literature. Based on this case and prior reports [5], syncope can be cured by multiple management, including: aggressive infection control, increased fluid intake, abdominal binder and compression stocking application during mobilization, slow transfer protocols with gradated torso elevation, low‐dose metoprolol (12.5 mg twice daily), and systematic rehabilitation.

Proactive management should address nutritional deficiencies and medication adverse effects. Patient education is crucial, including avoidance of abrupt positional changes, implementation of paced transfers during mobilization, nutritional adequacy maintenance, and moderate increases in water and salt intake [5, 12, 17, 18]. Rehabilitation must be integrated into protocols, initiating with supine aerobic and resistance exercises before progressively advancing to upright posture training [19, 20]. Pharmacologically, beta‐adrenergic blockers (e.g., metoprolol) demonstrate probable efficacy [5, 21].

We present this case to enhance understanding of post‐SCI POTS and to provide critical management strategies for recurrent syncope.

## 8 Conclusions

Post‐SCI orthostatic syncope with sinus tachycardia in the absence of OH should raise suspicion for POTS. Syncope may emerge as the predominant manifestation, potentially lacking characteristic symptoms associated with this condition. Infections are significant exacerbating factors. HRV and SSR tests may help elucidate the underlying pathogenesis. Management of POTS requires a multidisciplinary approach, integrating patient education, nursing care, pharmacotherapy, and structured rehabilitation.

## Author Contributions

**Yonghong Liu:** writing – original draft. **Boyan Fang:** writing – review and editing. **Qiaoxia Zhen:** conceptualization, writing – review and editing.

## Funding

The authors have nothing to report.

## Ethics Statement

The authors have nothing to report.

## Consent

Written informed consent was obtained from the patient to publish this report.

## Conflicts of Interest

The authors declare no conflicts of interest.

## Data Availability

The data that support the findings of this study are available on request from the corresponding author. The data are not publicly available due to privacy or ethical restrictions.
